# Ellagic Acid and Schisandrins: Natural Biaryl Polyphenols with Therapeutic Potential to Overcome Multidrug Resistance in Cancer

**DOI:** 10.3390/cells10020458

**Published:** 2021-02-21

**Authors:** Sabesan Yoganathan, Anushan Alagaratnam, Nikita Acharekar, Jing Kong

**Affiliations:** 1 Department of Pharmaceutical Sciences, College of Pharmacy and Health Sciences, St. John’s University, 8000 Utopia Parkway, Queens, NY 11439, USA; anushan.alagaratnam17@my.stjohns.edu (A.A.); nikita.acharekar14@my.stjohns.edu (N.A.); jing.kong13@my.stjohns.edu (J.K.); 2 Department of Chemistry, St. John’s College of Liberal Arts and Sciences, St. John’s University, 8000 Utopia Parkway, Queens, NY 11439, USA

**Keywords:** anticancer drugs, cancer, multidrug resistance, polyphenols, ellagic acid, schisandrin, quercetin, atropisomers, biaryl natural products

## Abstract

Multidrug resistance (MDR) is one of the major clinical challenges in cancer treatment and compromises the effectiveness of conventional anticancer chemotherapeutics. Among known mechanisms of drug resistance, drug efflux via ATP binding cassette (ABC) transporters, namely P-glycoprotein (P-gp) has been characterized as a major mechanism of MDR. The primary function of ABC transporters is to regulate the transport of endogenous and exogenous small molecules across the membrane barrier in various tissues. P-gp and similar efflux pumps are associated with MDR because of their overexpression in many cancer types. One of the intensively studied approaches to overcome this mode of MDR involves development of small molecules to modulate P-gp activity. This strategy improves the sensitivity of cancer cells to anticancer drugs that are otherwise ineffective. Although multiple generations of P-gp inhibitors have been identified to date, reported compounds have demonstrated low clinical efficacy and adverse effects. More recently, natural polyphenols have emerged as a promising class of compounds to address P-gp linked MDR. This review highlights the chemical structure and anticancer activities of selected members of a structurally unique class of ‘biaryl’ polyphenols. The discussion focuses on the anticancer properties of ellagic acid, ellagic acid derivatives, and schisandrins. Research reports regarding their inherent anticancer activities and their ability to sensitize MDR cell lines towards conventional anticancer drugs are highlighted here. Additionally, a brief discussion about the axial chirality (i.e., atropisomerism) that may be introduced into these natural products for medicinal chemistry studies is also provided.

## 1. Introduction

**Figure 1:**
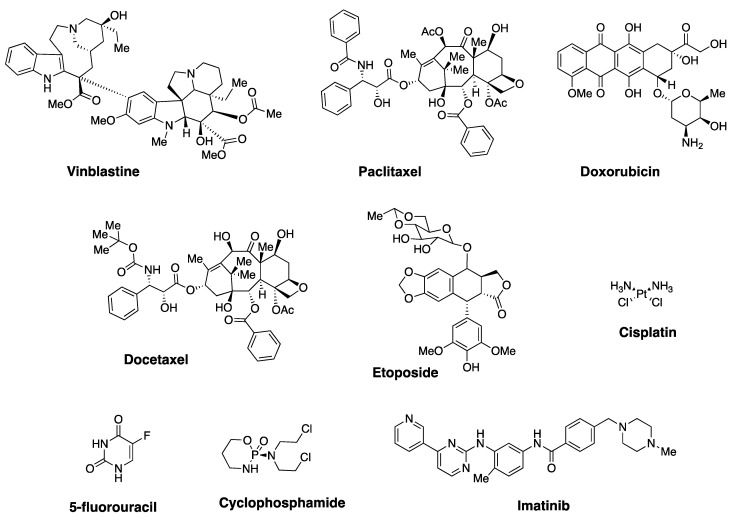
Structures of selected examples of clinically used anticancer drugs.

Cancer continues to be a major public health burden and places significant stress on global economy. Despite being a non-communicable disease, it is the second leading cause of death in the United States and in Europe [1]. On a global scale, one in six deaths is linked to cancer, and about 70% of deaths occur in low- and middle-income countries [1]. In 2018, approximately 9.6 million people died from various types of cancer [2]. Among the many different therapeutic interventions available, chemotherapeutics continue to be one of the primary choices for treating various types of metastatic cancer [3,4]. Vinblastine, paclitaxel, doxorubicin (Dox), docetaxel, etoposide, cisplatin, 5-fluorouracil (5-FU), cyclophosphamide, and imatinib are some of the widely used drugs for the treatment of cancer (Figure 1) [3,5]. These agents are natural products, natural product derivatives, or synthetic molecules, and have been developed to target different cellular pathways. Although many anticancer agents are readily available, drug resistance renders these conventional chemotherapeutics ineffective during cancer therapy.

**Figure 2:**
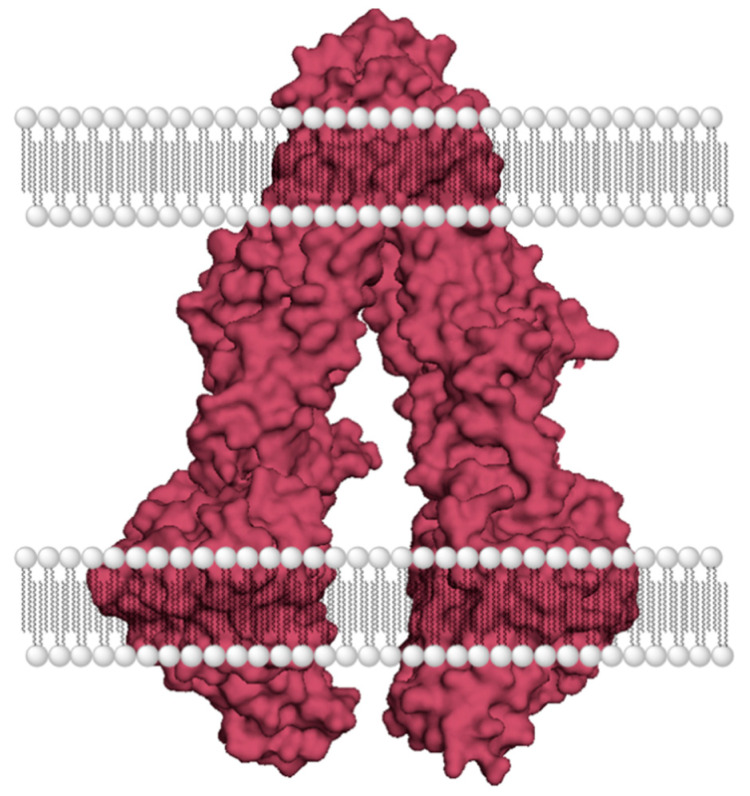
A crystal structure image of P-gp (PDB: 3G5U) [23].

During the course of a treatment regimen, cancer cells transform and develop resistance to these chemotherapeutics [6,7]. If a specific type of cancer exhibits drug resistance to a broader selection of drugs, the phenomenon is generally referred to as development of multidrug resistance (MDR) [7,8]. Some of the ways cancer cells develop drug resistance include (a) heightened DNA repair, (b) reduced drug uptake, (c) enhanced drug efflux, (d) mutation of drug targets, (e) changes in the inherent apoptotic process, and (f) increased drug metabolism [8]. As an example, MDR to paclitaxel has been attributed to increased levels of proteins such as mitogen-activated protein kinases (MAPKs), protein kinase B (PKB or Akt), and nuclear factor-κB (NF-κB), and overexpression of a type of ATP-binding cassette (ABC) transporter, referred to as P-glycoprotein (P-gp) [9,10]. Drug efflux mediated by ABC transporters has been identified as one of the major mechanisms of MDR for several classes of anticancer drugs, including etoposide, Dox, daunorubicin, vincristine, vinblastine, mitomycin C, and mitoxantrone, to name a few [11,12,13,14,15]. ABC transporters are a large family of membrane channels that regulate the movement of molecules of different sizes and chemical properties [16]. These transporters are found on the epithelial surface of various tissues, including brain, gastrointestinal tract, liver, renal tubules, adrenal cortex, and placenta [17]. They play a crucial role in the absorption, distribution, and excretion of various substances, including xenobiotics and endogenous molecules. P-gp, which is also referred to as MDR protein-1 (MDR1 or ABCB1) is the most studied ABC transporter and associated with MDR [12,18,19]. Two other ABC transporters responsible for MDR in cancer cells are MDR-associated protein 1 (MRP1 or ABCC1) and breast cancer resistance protein (BCRP or ABCG2) [12,20,21]. P-gp is a dimeric membrane glycoprotein, and the two halves exhibit about 43% sequence homology. Both halves of P-gp form a highly hydrophobic transmembrane domain (TMD), which contains the substrate-binding region. A well-accepted mechanism of drug transport involves an inward opening of TMDs to allow substrate binding and, subsequently, an outward opening of the TMDs to release the substrate during the process of drug efflux. ATP binds to the cytoplasmic nucleotide-binding domain (NBD) and successively undergoes hydrolysis to provide the energy needed for the transporter activity [16,22]. P-gp and similar efflux pumps have a large substrate-binding region within the TMDs, which allows the transport of a wide-range of substrates. A crystal structure image below (PDB: 3G5U) shows the dimeric structure, and the inward opening of P-gp (Figure 2) [23]. The large size and lack of substrate specificity enable P-gp to efflux several anticancer drugs out of cancer cells, despite their size and polarity. Due to a reasonable sequence homology between human P-gp and mouse P-gp, the structure of mouse P-gp (PDB: 6FN1) is often used as a homology model during drug discovery efforts [24,25].

Since the discovery of P-gp, MRP-1, and BCRP, and their connection to MDR in cancer, various approaches have been explored to overcome the drug efflux-linked therapeutic limitation [26,27]. One of the widely investigated and highly promising approaches involves the development of small molecules, including natural products, as potent modulators of such drug efflux pumps [28,29,30,31]. The rationale is that effective inhibitors of P-gp, when administered in combination with anticancer drugs, would increase the intracellular drug concentration and reverse MDR [32]. Based on this hypothesis, several generations of P-gp inhibitors have been investigated [26,27,28,29,30,31]. Cyclosporin A, a natural product and a first generation P-gp inhibitor, was moved up to phase III clinical trials but failed due to adverse effects [33]. First and second generation P-gp inhibitors were developed based on the mechanistic evidence that P-gp inhibition would enhance anticancer therapy. However, lead compounds from the initial phase of investigations were unsuccessful due to significant dose limiting toxicity profiles and cardiac complications. More recently, medicinal chemistry efforts led to the discovery of more promising third generation P-gp inhibitors, including tariquidar, zosuquidar, elacridar, and laniquidar [34,35]. These highly selective and potent agents advanced to clinical trials with high hope and promise because of their tolerable safety profile. However, dose limiting adverse effects in some cases, and low objective response rate in metastatic cancer prevented these leads from advancing further. Based on the proposed mode of action, the third generation P-gp inhibitors, such as tariquidar and elacridar, are acting directly on P-gp to modulate its activity, which is a similar mode of inhibition for many earlier generation P-gp inhibitors. Currently reported compounds inhibit P-gp activity by either modulating its ATPase activity or competitively binding to the substrate binding sites. Polyphenols, on the contrary, have the ability to overcome P-gp based MDR via direct and indirect mechanisms. The indirect mode of action relates to polyphenols’ ability to target various signaling pathways that are linked to P-gp expression. Moreover, polyphenols are structurally much different than the third generation P-gp inhibitors, and provide a new chemical space for exploration. Since these natural products have diverse functional groups, a semi-synthetic approach could provide easy access to new structural analogs. Polyphenols are also widely known for their antioxidant and anti-inflammatory properties, and it is worth noting that such inherent beneficial effects may provide added advantages during the exploration of these natural products [27].

Since the P-gp inhibitors developed to date have not provided a successful path forward, there is a continuing interest in identifying new classes of P-gp inhibitors as more efficacious clinical candidates [36,37,38,39]. Towards this direction, polyphenolic natural products have emerged as promising scaffolds for drug discovery efforts [40,41]. Furthermore, polyphenols and their analogs may be developed to be used in combination with one or more of the most successful third generation P-gp inhibitors to overcome MDR in cancer.

## 2. Natural Polyphenols and Their Anticancer Properties

**Figure 3:**
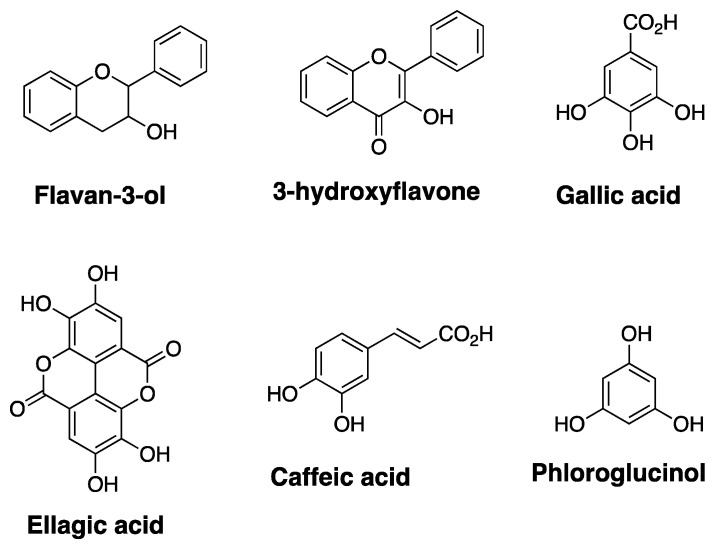
Structures of common polyphenolic core-scaffold.

Natural products are highly effective and structurally complex secondary metabolites with a wide range of medicinal properties. Nature has optimized the molecular scaffold of natural products by incorporating various functional groups, enabling natural products to effectively bind to chiral binding sites within biological targets. Natural products from microorganisms, plants, and animals have provided some of the most effective drugs and drug leads to date [42,43,44,45,46]. Once new classes of natural products are discovered, scientists employ chemical and biological approaches to transform natural products into pharmacologically optimized structures for therapeutic applications [47,48,49,50]. With the successful development of several natural products and their derivatives as effective anticancer drugs, there is a continuing interest to discover new and more effective anticancer natural products. Polyphenols are a class of highly oxygenated aromatic compounds and have emerged as promising drugs with broad spectrum of bioactivity [51]. Many of the plant-derived polyphenols can be classified into sub-categories based on their core-structure. Some of the core structures found within various polyphenols, such as flavan-3-ol, 3-hydroxyflavone, gallic acid, ellagic acid, caffeic acid, and phloroglucinol are highlighted in Figure 3.

**Figure 4:**
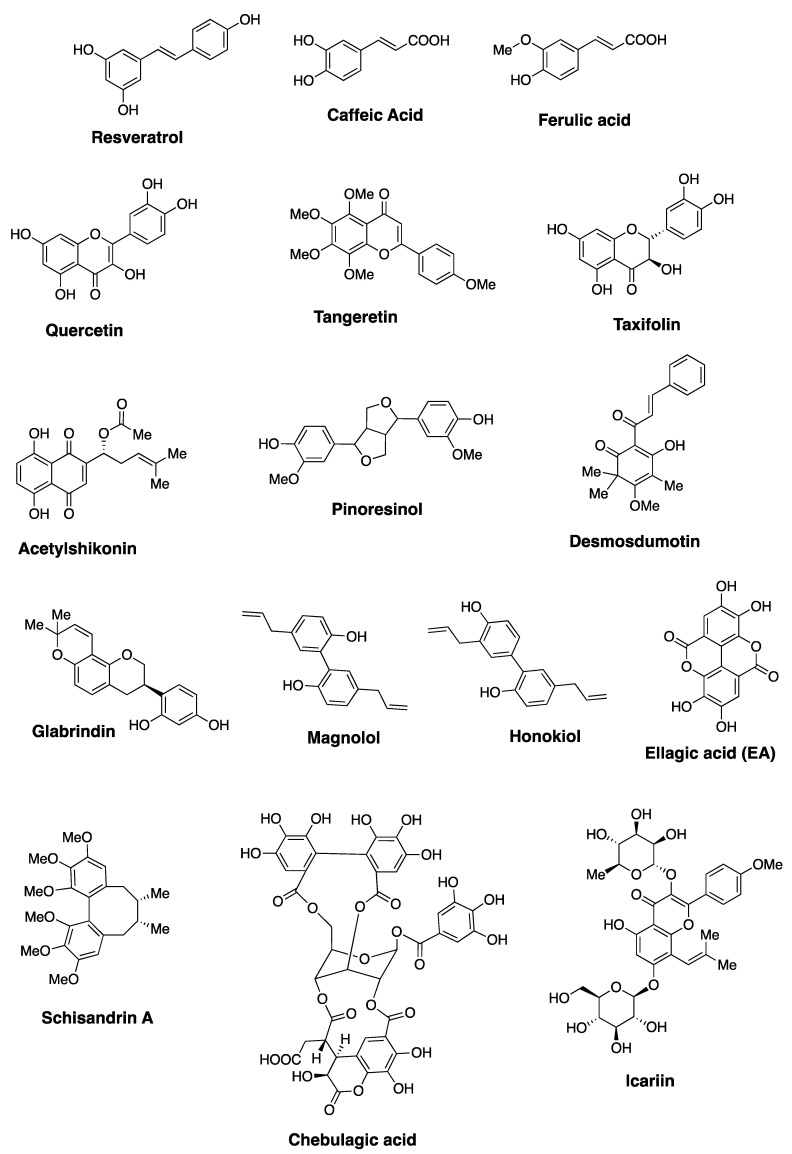
Structures of selected examples of biologically active polyphenols.

More than 800 polyphenolic compounds are broadly described as flavonoids and they exhibit a range of biological activities, including anticancer activity [51]. The structures of selected examples of structurally unique and biologically important polyphenols are shown in Figure 4. Among the various polyphenols investigated for anticancer activities and their ability to modulate P-gp function, quercetin has gained considerable attention [30]. Although quercetin is not the major focus of this review, its anticancer potential is briefly discussed here. Quercetin exhibits anti-inflammatory/antioxidant activity as it is capable of quenching free radicals. It exhibits anticancer activity via various mechanisms, including cell cycle arrest, apoptosis and/or alteration of various signaling pathways [52,53]. A substantial amount of literature reports are available on the anticancer activity of quercetin against several cancer types, including breast, lung, liver, pancreatic, colon, cervical, ovarian, and kidney cancers. For a comprehensive review on the anticancer properties of quercetin, readers are directed to a review article published by Mubarak and coworkers [53]. Quercetin has been shown to enhance the therapeutic outcome of cisplatin in a synergistic fashion, and sensitivity of breast cancer cell lines to Dox [54,55]. In addition to showing cytotoxicity, quercetin and similar chromones have been identified as modulators of P-gp, MRP-1, and BCRP [20,56,57]. It is important to note that conversion of the hydroxyl in these natural products to the corresponding methyl ethers improved the inhibitory activity towards MDR efflux pumps. Current understanding of the SAR of quercetin provides a strong foundation to further develop similar polyphenols into promising anticancer agents.

### 2.1. Ellagic Acid

Ellagitannins represent a diverse class of polyphenolic natural products with remarkable structural complexity [58,59]. The various members of ellagitannins are generally glycosyl esters of ellagic acid and/or gallic acid motifs. Some members also contain flavone motifs as part of the structure, referred to as flavano-ellagitannins. Ellagic acid (EA), a component of ellagitannins, is a biaryl polyphenol where two gallic acid motifs are oxidatively coupled via a carbon-carbon bond to join the two aryl rings. The biosynthetic modification provides a structurally unique biaryl motif that has the potential to exhibit axial chirality, referred to as ‘atropisomerism’ [60,61]. EA is generated via a hydrolysis reaction under acidic or basic pH, and the ester linkages within ellagitannins are hydrolyzed to generate the acyclic carboxylic acid derivative of EA. This intermediate readily undergoes an intramolecular lactonization to generate EA (Scheme I). As a biologically active secondary metabolite, EA and ellagitannins exhibit beneficial effects towards various diseases, including microbial infection, cancer, and neurodegenerative diseases, mainly due to their antioxidant properties [62]. For example, chebulagic acid (Figure 4), an ellagitannin, synergistically enhances the anticancer activity of Dox, and overcomes MDR-1 mediated drug resistance in HepG2 cell line [63]. The biological properties of EA have been extensively reviewed in the literature and readers are directed to published work on this topic [62,64]. The presence of poly-oxygenated aryl rings allows EA to quench free radicals, making it a highly effective antioxidant and cytoprotective agent. EA has been reported to provide a protective effect against reactive oxygen species in biological environments [65]. One of the major pharmacological limitations of EA relates to its low solubility in water, which leads to significantly reduced bioavailability. Chemical modification of EA to enhance solubility and bioactivity is of high interest to medicinal chemists. Additionally, gut bacteria are known to metabolize EA into urolithins, which have better bioavailability compared to EA. The rate of metabolism of EA by gut bacteria and the levels of urolithins may be attributed to discrepancies in pharmacological outcomes observed from in vitro versus in vivo studies [66].

**Figure 5:**
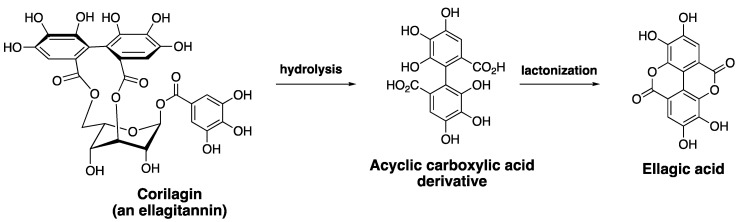
Ellagic acid and structurally similar derivatives.

With regards to anticancer activity, EA showed cytotoxicity against A549 cell line, and the cytotoxicity was linked to inhibition of sphingosine kinase 1 (SphK1). Computational methods and kinase inhibition assays were used to support SphK1 inhibition as a mechanism of cytotoxicity. The binding is proposed to occur within the ATP binding pocket of SphK1 [67]. In a recent study, Ceci et al. reported that EA reduced the invasive nature of bladder cancer through VEGF-mediated pathways by testing EA against four different human bladder cancer cell lines (T24, UM-UC-3, 5637 and HT-1376) [68]. EA also exhibited selective activity against A549, HepG2, and MCF-7 cell lines, compared to HEK293 cell line [69,70,71]. The mechanism of cytotoxicity against A549 is linked to inhibition of PI3K/Akt pathway [69], and pyruvate dehydrogenase kinase 3 (PDK3) activity [70]. EA was identified as an inhibitor of integrin-linked kinase (ILK) in the breast cancer cell line, MCF-7 as well [71]. Recently, EA has attracted the attention of researchers who are developing drug leads to overcome MDR in cancer. Along with a series of 3,4-dihydroisocoumarins, Sachs et al. evaluated the activity of EA against A549, HCT-15 (expresses high levels of P-gp), and MCF-7/MX (overexpresses BCRP) cell lines. EA showed an IC_50_ value of >50 μΜ against all three cell lines, where the isocoumarin derivative-1 (Figure 5) showed selective toxicity towards A549, and has an IC_50_ of > 100 μΜ against HCT-15 and MCF-7/MX [72]. Moreover, the isocoumarin derivative-1 showed a dose-dependent inhibition of P-gp and BCRP. Current literature data support that EA has considerable potential as an anticancer lead, and medicinal chemistry could improve its physiochemical properties and perhaps provide a better understanding of its ability to overcome P-gp mediated MDR.

### 2.2. Schisandrins and Related Lignans

**Figure 6:**
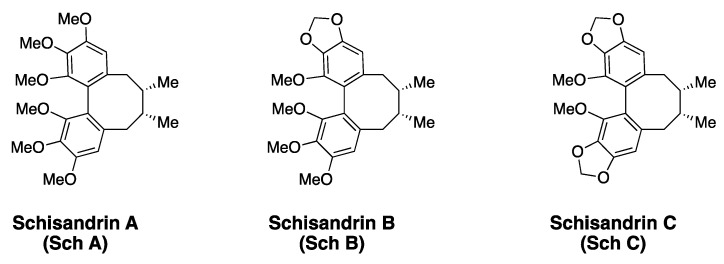
Structures of schisandrin A, B and C.

Schisandrins are an important class of lignans and are characterized by the presence of the ‘dibenzocyclooctadiene’ skeleton. These natural products were isolated from the plant species *Schisandra chinesis*, and the extract from the Schisandraceae family was used as part of traditional Chinese medicine [73]. The biologically active components in the natural remedy were identified as dibenzocyclooctadienes and exhibited a wide range of bioactivity, including antioxidant, anticancer, and hepatoprotective effects [74]. The dibenzocyclooctadiene class polyphenols include several members, and are distinguished by the identity of chemical groups attached to the biaryl-rings and the cyclooctadiene ring [74]. The biaryl motif of schisandrins is structurally similar to EA and contains the 3,4,5-trioxygenated benzene ring. However, the cyclooctadiene ring provides more flexibility to the biaryl motifs in schisandrins, and a chance to exhibit atropisomerism. Since it is impossible to discuss all members of the ‘dibenzocyclooctadiene’ class natural products within this review, the scope of this review is limited to schisandrins, and their anticancer properties. The three prominent members of schisandrins reported to date are schisandrin A (Sch A), schisandrin B (Sch B) and schisandrin C (Sch C). Sch A is a per-methylether compound, while Sch B and Sch C have a 1,3-benzodioxole group as part of the biaryl ring (Figure 6). Other derivatives of schisandrins are also known in the literature, where the cyclooctadiene ring is oxygenated, but those members are not discussed here [74,75].

Sch A exhibits promising anticancer activities against thyroid, colorectal, breast, and lung cancer cell lines [76,77,78,79,80]. Xu et al. report that Sch A is active against two triple-negative breast cancer cell lines, MDA-MB-231 and BT-549 in vitro and in xenograft mouse models. Sch A induced cell cycle arrest and apoptosis via Wnt/ER signaling pathway [79]. Sch A also inhibited the proliferation of colorectal cancer cell lines (RKO, DLD-1, SW620, and SW480), causing cell cycle arrest and apoptosis [78]. Chen et al. report that the mechanism of cytotoxicity against these cell lines is linked to heat shock proteins function [78]. Interestingly, Sch A was reported as a chemosensitizer and improved the activity of conventional anticancer drugs against MDR cell lines. In another report, Sch A rescued the anticancer activity of gefitinib towards a gefitinib-resistant cell line (HCC827/GR) by inhibiting IKKβ/NF-κB signaling [81]. Furthermore, Sch A reversed P-gp mediated Dox resistance in MCF-7/Dox cell line. It also inhibited the NF-κB and Stat3 signaling pathways to rescue the activity of Dox [82]. A recent report by Kong et al. showed that Sch A improved the activity of 5-FU against two colorectal cancer cell lines, HCT-116 and SW480. The report indicates that sensitization of HCT-116 and SW480 cell lines towards 5-FU is through PI3K/Akt and NF-κB pathways [83].

Sch B has been extensively investigated for its anticancer properties and its ability to overcome MDR in cancer [84,85,86,87,88,89,90,91,92]. A recent study reported that Sch B was active against three different triple negative breast cancer cell lines (MDA-MB-231, BT-549, and MDA-MB-468) and inhibited cell growth via cell cycle arrest and apoptosis. The mechanism of cytotoxicity relates to Stat3 inactivation [86]. When a hepatic carcinoma cell line (SMMC7721) and a breast cancer cell line (MCF-7) were treated with Dox and Sch B together, Sch B considerably enhanced the anticancer effect. It is worth noting that Sch B did not increase Dox-induced apoptosis in rat cardiomyocytes and human fibroblasts. Li et al. reported that the enhanced cytotoxicity was due to caspase-9 activation, and less likely to be via P-gp or other efflux pump inhibition [89]. Researchers have explored the potential of Sch B to be a useful inhibitor of P-gp as well. Based on a report by Wang et al., Sch B sensitized a Dox-resistant breast cancer cell line (MCF-7/ADR) and ovarian cancer cell line (A2780/Dox) by inhibiting P-gp expression and P-gp mediated efflux of Dox [90]. Sch B alone and in combination with Dox showed a concentration-dependent inhibition of P-gp expression, as well as P-gp activity [90]. A report by Sun et al. confirms that Sch B not only inhibits P-gp activity but also MRP-1 mediated drug efflux [91]. Sch B reversed MRP-1 mediated drug resistance in HL60/ADR and HL60/MRP cell lines, and sensitized these cell lines towards daunorubicin. Compared to probenecid, a known MRP-1 inhibitor, Sch B showed noticeably stronger potency [91]. Hu and coworkers also showed that Sch B reversed P-gp mediated drug resistance in K562/ADR, MCF-7/ADR, Bcap37/ADR, and KBv200 cell lines [92], Several conventional anticancer drugs were evaluated in combination with Sch B or verapamil against these cell lines, and Sch B showed promising drug reversal data. As per the authors, Sch B has an advantage over verapamil because Sch B did not exhibit a similar toxicity profile as verapamil [92]. Based on literature evidence, Sch B has emerged as a structurally novel natural product that modulates P-gp and similar efflux pump mediated MDR in cancer [91,92]. Although Sch C shares structural similarities to Sch A and Sch B, limited studies have been done to evaluate its anticancer potential. Sch C, also referred to as wuweizisu C, was initially identified as a hepatoprotective agent [93]. Lu et al. [94] isolated and reported the selective anticancer activity of Sch C towards hepatocellular carcinoma cells (Bel-7402), compared to a breast cancer cell line (Bcap37) and a nasopharyngeal carcinoma cell line (KB-3-1). Sch C also exhibited anticancer activity against human leukemia U937 cells, where it induced cell cycle arrest and apoptosis [95]. Taken together, Sch A, Sch B, and Sch C are useful polyphenols with a ‘biaryl’ scaffold and show considerable potential as anticancer agents and modulators of P-gp activity (Table 1). It is important to note that these natural products exhibit anticancer activity via various biochemical mechanisms. By altering the signaling pathways that are linked to tumorigenesis, and sensitizing MDR-cell lines towards anticancer drugs, schisandrins exhibit promising anticancer potential. Medicinal chemists are interested in synthesizing structural analogs of schisandrins to improve their anticancer potential [96,97]. Current efforts have focused on understanding the SAR related to the cyclooctadiene ring. However, the biaryl scaffold in this class of natural products provides another useful avenue for structure diversification of schisandrins.

## 3. Atropisomerism in Drug Discovery

**Figure 7:**
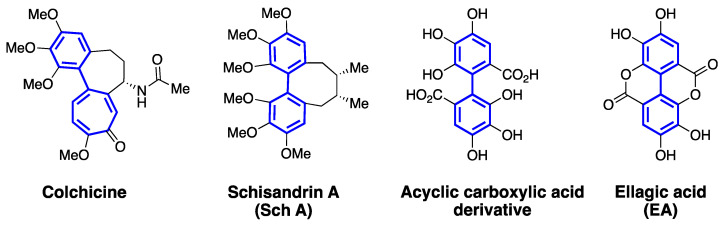
Structures of natural products that exhibit an axis of chirality along the biaryl motifs.

Chirality of molecules often plays an important role in their biological activity. There are many examples of drugs to illustrate that for a mixture of enantiomers, one isomer exhibits desired biological activity, and the other isomer is either inactive or exhibits adverse effects. Classically, chirality of a molecule is linked to stereocenters (i.e., enantiomers or diastereomers), and racemization of such molecules involves bond breaking or bond forming steps. Atropisomerism typically arises via rotation along a bond that connects two sp2-hybridized atoms, and isomers with opposing optical properties are obtained. Biaryl systems are classically known for their ability to exhibit atropisomerism and have been well explored for structure, stability, and function. 1,1′-Bi-2-naphthol (BINOL) is a common example in chemistry, which exhibits atropisomerism, and extremely difficult to racemize at room temperature. Within the biaryl-containing small molecules, steric hindrance from substituents on the aryl ring limits the rotation of two motifs along a chiral axis. If the size of the substituents on the aryl rings are small, there is free rotation along the chiral axis and the isomers racemize freely at room temperature. Atropisomerism in drug discovery has been widely accepted due to the presence of one or more axial chirality in a large percentage of clinically used drugs [98,99,100]. Although rapidly interconverting atropisomers are often characterized as achiral entities, it is important to recognize that such molecules bind to a biological target as one type of isomer [98,99]. Moreover, similar to enantiomers, atropisomers can exhibit different binding affinity towards an intended target and toxicity profile [100,101]. Colchicine (Figure 7) is a natural product and exhibits atropisomerism. The bioactive and stable isomer of colchicine is identified as (R_a_, 7*S*)-colchicine. The stability of this particular atropisomer is attributed to the ‘cycloheptadiene’ ring and the acetamido group at 7-position [61,102]. It is interesting to note that the biaryl-motifs of schisandrins (Figure 6 and Figure 7) also share a similar mode of conformational stability due to the presence of an cyclooctadiene ring. Based on the understanding of biaryl-containing atropisomeric molecules, we hypothesize that EA, schisandrins and similar polyphenols can be suitably derivatized to generate stable atropisomers.

EA, as a bis-lactone, is a planar molecule and has substantial limitations in terms of physiochemical properties and anticancer properties. On the other hand, the acyclic carboxylic acid derivative of EA (Figure 7) has an atropisomeric axis and has the ability to rotate around the biaryl axis. Literature reports indicate that a hydroxyl on the carbon adjacent to the atropisomeric axis is not large enough to restrict the interconversion of the isomers. It is very possible to modify the hydroxyls and/or carboxyl groups on the aryl rings of EA with a suitable substituent to increase the rotational barrier and generate atropisomeric derivatives. Schisandrins, on the other hand, are not planar, and likely to exhibit atropisomerism. Based on the structure of colchicine, one can design atropisomerically pure structural analogs of schisandrins through modification of the cyclooctadiene ring and/or the methoxy group adjacent to the biaryl axis. Since EA and schisandrins show promise as anticancer agents and promising inhibitors of P-gp, MRP-1, and BCRP, their therapeutic potential can be further explored by studying stable atropisomers. In comparison to traditional chemical approaches available to rigidify the flexible or more planar polyphenol scaffolds, rigidification of an interconverting atropisomeric axis is a synthetically more viable option for drug discovery efforts.

## 4. Conclusions

Polyphenolic natural products represent a chemically unique class of molecules as potential anticancer agents. This review summarizes the chemical structure and anticancer activities of a class of structurally similar polyphenols, EA and schisandrins. They share a 3,4,5-trioxygenated biaryl scaffold as the core structure, yet schisandrins exhibit slightly more structural complexity. These natural products exhibit inherent anticancer activities against various cancer types and through different modes of action. Moreover, they have emerged as promising leads to address the challenges related to MDR in cancer. Schisandrins improve the anticancer activities of conventional anticancer drugs against MDR cell lines by various biochemical mechanisms. One of the major advantages of polyphenols relates to their ability to modulate drug efflux by P-gp and similar transporters, and their expression at the transcription level. Therefore, polyphenols hold promise as an important class of modulators of MDR in cancer. Since the atropisomeric properties of these polyphenols have not been explored in detail, this review highlights the possibility of generating conformationally rigid atropisomers of EA and schisandrins for medicinal chemistry evaluation.

## Figures and Tables

**Table 1:** Summary of anticancer activities of EA and schisandrins.

Polyphenol	Cancer Cell Line	Tumor Type	Anticancer Mechanism	Ref.
**Ellagic acid**	A549	non-small cell lung cancer	SphK1 inhibition	[67]
			PI3K/Akt pathway and PDK3 activity	[69,70]
	T24, UM-UC-3, 5637, HT-1376	bladder cancer	VEGF-mediated pathway	[68]
	HepG2	liver cancer	PI3K/Akt pathway and PDK3 activity	[69,70]
	MCF-7	breast cancer	PI3K/Akt pathway and PDK3 activity	[69,70]
			integrin-linked kinase (ILK) inhibition	[71]
**Sch A**	MDA-MB-231, BT-549	breast cancer	cell cycle arrest/apoptosis via Wnt/ER pathway	[79]
	RKO, DLD-1, SW620, SW480, HCT-116	colorectal cancer	cell cycle arrest/apoptosis; cytotoxicity is via heat shock protein function	[78]
			increased sensitivity to 5-FU via PI3K/Akt and NF-kB signaling	[83]
	HCC827/GR	Gefitinib-resistant lung cancer	inhibition of IKKb/NF-kB signaling	[81]
	MCF-7/Dox	Dox-resistant breast cancer	reversal of P-gp mediated MDR via Stat3 and NF-kB pathway	[82]
**Sch B**	MDA-MB-231, BT-549, MDA-MB-468	breast cancer	cell cycle arrest/apoptosis via Stat3 pathway	[86]
	SMMC7721	liver cancer	enhanced the anticancer activity of Dox	[89]
	MCF-7	breast cancer	enhanced the anticancer activity of Dox	[89]
	MCF-7/ADR	Dox-resistant breast cancer	decreased P-gp expression and P-gp mediated efflux	[90,92]
	A2780/Dox	Dox-resistant ovarian cancer	decreased P-gp expression and P-gp mediated efflux	[90]
	HL60/ADR, HL60/MRP, K562/ADR	drug-resistant leukemia	sensitized the cells to daunorubicin; reversed P-gp mediated drug resistance	[91,92]
	Bcap37/ADR	drug-resistant breast cancer	reversed P-gp mediated drug resistance	[92]
	KBv200	drug-resistant epidermoid carcinoma	reversed P-gp mediated drug resistance	[92]
**Sch C**	Bel-7402	liver cancer	undisclosed mechanism	[94]
	Bcap37	breast cancer	undisclosed mechanism	[94]
	KB-3-1	nasopharyngeal cancer	undisclosed mechanism	[94]
	U937	leukemia	cell cycle arrest and apoptosis	[95]

## Data Availability

Data sharing not applicable.
